# Attenuated HIV-1 Nef But Not Vpu Function in a Cohort of Rwandan Long-Term Survivors

**DOI:** 10.3389/fviro.2022.917902

**Published:** 2022-06-16

**Authors:** Gisele Umviligihozo, Jaclyn K. Mann, Steven W. Jin, Francis M. Mwimanzi, Hua-Shiuan A. Hsieh, Hanwei Sudderuddin, Guinevere Q. Lee, Helen Byakwaga, Conrad Muzoora, Peter W. Hunt, Jeff N. Martin, Jessica E. Haberer, Etienne Karita, Susan Allen, Eric Hunter, Zabrina L. Brumme, Mark A. Brockman

**Affiliations:** 1 Faculty of Health Sciences, Simon Fraser University, Burnaby, BC, Canada; 2 HIV Pathogenesis Programme, University of KwaZulu-Natal, Durban, South Africa; 3 Department of Molecular Biology and Biochemistry, Simon Fraser University, Burnaby, BC, Canada; 4 British Columbia Centre for Excellence in HIV/AIDS, Vancover, BC, Canada; 5 Department of Medicine, Weill Cornell Medical College, New York, NY, United States; 6 Department of Community Health, Mbarara University of Science and Technology, Mbarara, Uganda; 7 Department of Medicine, University of California, San Francisco, CA, United States; 8 Center for Global Health, Massachusetts General Hospital, Boston, MA, United States; 9 Department of Medicine, Harvard Medical School, Boston, MA, United States; 10 Centre for Family Health Research, Kigali, Rwanda; 11 Department of Pathology and Laboratory Medicine, Emory University, Atlanta, GA, United States; 12 Emory Vaccine Center at Yerkes National Primate Research Center, Atlanta, GA, United States

**Keywords:** HIV non-progressors, viral accessory proteins, immune evasion, pathogenesis, downregulation, CD4, tetherin, HLA

## Abstract

HIV-1 accessory proteins Nef and Vpu enhance viral pathogenesis through partially overlapping immune evasion activities. Attenuated Nef or Vpu functions have been reported in individuals who display slower disease progression, but few studies have assessed the relative impact of these proteins in non-B HIV-1 subtypes or examined paired proteins from the same individuals. Here, we examined the sequence and function of matched Nef and Vpu clones isolated from 29 long-term survivors (LTS) from Rwanda living with HIV-1 subtype A and compared our results to those of 104 Nef and 62 Vpu clones isolated from individuals living with chronic untreated HIV-1 subtype A from the same geographic area. *Nef* and *vpu* coding regions were amplified from plasma HIV RNA and cloned. The function of one intact, phylogenetically-validated Nef and Vpu clone per individual was then quantified by flow cytometry following transient expression in an immortalized CD4+ T-cell line. We measured the ability of each Nef clone to downregulate CD4 and HLA class I, and of each Vpu clone to downregulate CD4 and Tetherin, from the cell surface. Results were normalized to reference clones (Nef-SF2 and Vpu-NL4.3). We observed that Nef-mediated CD4 and HLA downregulation functions were lower in LTS compared to the control cohort (Mann-Whitney p=0.03 and p<0.0001, respectively). Moreover, we found a positive correlation between Nef-mediated CD4 downregulation function and plasma viral load in LTS and controls (Spearman ρ= 0.59, p=0.03 and ρ=0.30, p=0.005, respectively). In contrast, Vpu-mediated functions were similar between groups and did not correlate with clinical markers. Further analyses identified polymorphisms at Nef codon 184 and Vpu codons 60-62 that were associated with function, which were confirmed through mutagenesis. Overall, our results support attenuated function of Nef, but not Vpu, as a contributor to slower disease progression in this cohort of long-term survivors with HIV-1 subtype A.

## INTRODUCTION

The rate of disease progression following human immunodeficiency virus type 1 (HIV-1) infection can vary widely (1). In the absence of combination antiretroviral therapy (cART), symptoms of acquired immunodeficiency syndrome (AIDS) may appear within as little as one year or as long as ten years (or more) after infection (2, 3). Such heterogeneity has prompted studies of people living with HIV (PLWH) who display extreme protective phenotypes, as defined by prolonged maintenance of favorable clinical measures such as high CD4+ T cell counts and low plasma HIV-1 viral loads (pVL) in the absence of therapy (4, 5). The small subset of PLWH who remain healthy for many years in the absence of cART are variously known as long-term non-progressors, elite controllers, elite suppressors or long-term survivors (LTS), with somewhat overlapping definitions based on the specific classification criteria used (5).

Both host and viral genetic factors are associated with HIV non-progressor phenotypes (6). Host factors include polymorphisms in cellular genes required for HIV-1 replication such as the viral coreceptor CCR5 (7) as well as natural variation in immune genes including the Human Leukocyte Antigens (HLA) (8). Stochastic differences in the host immune response to infection such as the development of higher avidity and more cross-reactive antiviral T cells have also been described (9). Viral genetic variation influences HIV-1 set point viral load (10) and mutations that attenuate viral replication and pathogenesis contribute specifically: deletions or polymorphisms that impair the function of viral structural proteins such as Gag or Env, as well as those affecting accessory proteins such as Nef, Vpu or Vpr can influence disease outcome (11).

HIV-1 Nef and Vpu enhance viral pathogenicity through partially overlapping immune evasion functions (12-14). Both proteins downregulate the viral entry receptor CD4 from the cell surface, thereby shielding the infected cell from elimination by antibody-dependent cellular cytotoxicity (ADCC), which requires Env on the cell surface to be in its CD4-bound confirmation (15-17). Nef also prevents antigen presentation by internalizing HLA class I, while Vpu enhances virion release and protects infected cells from ADCC by internalizing Tetherin (12, 18, 19). Studies of humans and non-human primates have demonstrated that impairment of Nef or Vpu is associated with slower disease progression (20-22). Additionally, attenuated Nef or Vpu function has been reported in some non-progressors (23-26). Most studies however have focused only on HIV-1 subtype B and few reports have assessed both Nef and Vpu function in the same individuals. The relative roles of Nef and Vpu in the context of non-progression thus remain incompletely understood, particularly in the context of non-B subtypes.

In the present study, we analysed the sequence and *in vitro* function of autologous Nef and Vpu clones isolated from 29 LTS from Rwanda with chronic HIV-1 subtype A infection. Data were compared to an existing panel of 104 Nef and 62 Vpu clones isolated from individuals from the same geographic area with chronic HIV-1 subtype A infection. Our results indicate that impaired function of Nef, but not Vpu, is associated with slower disease progression in this cohort of African LTS.

## MATERIALS AND METHODS

### Study Participants and Ethics Approvals

LTS plasma samples were obtained from a longitudinal cohort of PLWH from Rwanda, which was established in 1986 by Projet San Francisco and the Centre for Family Health Research. Control plasma samples were obtained from participants of the Heterosexual Transmission [HT] study in Kigali, Rwanda. Previously-generated Vpu (27) and Nef (28) clones from participants of the Uganda AIDS Rural Treatment Outcomes [UARTO] study in Mbarara, Uganda were also included as controls. All participants provided written informed consent prior to enrolment. The present study was approved by the Rwanda National Ethics committee, the Emory University Institutional Review Board, and the Simon Fraser University Research Ethics Board.

### Amplification and Sequencing of *Nef and Vpu*

HIV-1 RNA was extracted from plasma using the NucliSENS EasyMag system (bioMérieux). The *nef* and *vpu* coding regions were amplified by reverse transcription-polymerase chain reaction (RT-PCR) using the Superscript III one-step RT-PCR system with Platinum *Taq* HiFi (Invitrogen) as described previously for Nef (28, 29) and Vpu (27). First-round amplicons were subjected to a nested second-round PCR reaction using the Expand High-Fidelity Plus PCR system (Roche) and primers containing AscI and SacII restriction enzyme sites to facilitate cloning. First-round *nef* primers, designed to accommodate HIV-1 subtype diversity, were: forward 5’- TAGCAGTAGCTGRGKGRACAGATAG-3’ (HXB2 nt 8683-8707); reverse 5’-TACAGGCAAAAAGCAGCTG CTTATATGYAG- 3’ (HXB2 nt 9536-9507). Second-round *nef* primers were: forward 5’-AGAGCACCGGCGCGCC**TCCACATACCTASAAGAATMAGACARG**- 3’, (HXB2 nt 8746-8772 in bold text; AscI restriction site is underlined); reverse 5 ‘ - GCCTCCGCGGATCGAT**CAGGCCACRCCTCCCTGGAAASKCCC**- 3’, (HXB2 nt 9474-9449, in bold text; SacII restriction site is underlined). First-round *vpu* primers were: forward, 5′-TTGGGTGYCRRCAYAGCAGRATAGG-3′ (HXB2 nt 5780-5804); reverse, 5′-ATRTGCTTTVGCATCTGATGCACARAATA-3′ (HXB2 nt 6407-6379). Second-round *vpu* primers were: forward, 5′-AGAGGGCGCGCC**ATCAARHTYCTVTAYCAAAGCAGTAAGTA**-3′; (HXB2 nt 6024-6052 in bold; AscI restriction site is underlined); reverse 5′-GCCTCCGCGGATCGAT**GGTACCCCATARTAGACHGTRACCCA**-3′ (HXB2 nt 6352-6327 in bold; SacII restriction site is underlined). Amplicons were Sanger sequenced on a 3730xl automated Genetic Analyzer (Applied Biosystems Inc.). Chromatograms were assembled using Sequencher v5.0.1 software (GeneCodes).

### Nef and Vpu Cloning

*Nef* and *vpu* amplicons were cloned into eukaryotic expression vectors as described (28, 30). Briefly, second-round products were purified using the E.Z.N.A Cycle Pure kit (Omega Bio-tek). Each *nef* or *vpu* amplicon was then cloned into a modified version of pSELECT-GFPzeo (*In vivo*Gen) that contained 5’ AscI and 3’ SacII sites, where expression is driven by a composite hEF1-HTLV promoter. pSELECT-GFPzeo also features an independent CMV/HTLV promoter driving the expression of GFP. The pSELECT-GFPzeo vector used for *vpu* cloning was further modified to include the HIV-1 Rev Responsive Element (RRE) sequence downstream of the multiple cloning site to enhance Vpu expression (pSELECT-RRE-GFP). Following ligation, DNA products were transformed into *E. cloni* 10G chemically competent cells (Lucigen) and plated on Luria-Bertani (LB) agar containing Zeocin. Colonies were isolated, grown in LB medium containing Zeocin, and plasmid DNA was purified using the E.Z.N.A Plasmid DNA Mini kit (Omega Bio-tek). Plasmid clones were validated by Sanger sequencing. Following phylogenetic authentication (see below), one intact *nef* and one intact *vpu* clone per participant was selected for *in vitro* functional analysis (Supplementary Figure 1).

### Phylogenetic Analysis and Subtype Determination

Alignments of intact clonal sequences and their original bulk plasma sequences were generated using HIV Align (www.hiv.lanl.gov/content/sequence/VIRALIGN/viralign.html) (31) and manually edited using Aliview (32). Maximum likelihood phylogenetic trees were inferred using using PhyML (33) and visualized using FigTree v1.4.4 (http://tree.bio.ed.ac.uk/software/figtree/) (34). HIV subtype classification was performed using the Recombinant Identification Program (RIP) (www.hiv.lanl.gov/content/sequence/RIP/RIP.html) (35), using a window size of 100 and confidence threshold of 95%.

### Flow Cytometry

To examine Nef function, 2.0 μg of pSELECT-Nef-GFP was transfected into 1 x 10^6^ CEM T cells resuspended in 50 μl Opti-MEM-I medium (Life Technologies) by electroporation using a Bio-Rad GenePulser MxCell instrument (single 25-ms square-wave pulse at 250 V, 2,000 μF, infinite Ω). All electroporations were performed in a 96-well plate (Bio-Rad cat #1652681). To examine Vpu function, 1.5 μg of pSELECT-Vpu-RRE-GFP plus 2.0 μg of pSELECT-Rev was transfected into 0.5 x 10^6^ CEM cells using the same protocol. Transfected cells were resuspended in 400 μl RPMI 1640 medium, supplemented with 2 mM l-glutamine, 1,000 U/ml penicillin and 1 mg/ml streptomycin (all from Sigma-Aldrich) plus 10% fetal bovine serum (Life Technologies) and incubated for 24 h at 37°C with 5% CO_2_. For Nef, 0.25 x 10^6^ cells were stained with allophycocyanin (APC) labeled anti-human CD4 (clone RPA-T4; BD Biosciences) and phycoerythrin (PE) labeled anti-human HLA-A*02 (clone BB7.2; BioLegend) antibodies. For Vpu, 0.25 x 10^6^ cells were stained with allophycocyanin (APC) labeled anti-human CD4 (clone RPA-T4; BD Biosciences) and phycoerythrin (PE) labeled anti-human CD317/BST2/tetherin (clone RS38E; BioLegend) antibodies. Stained cells were incubated at 4°C for 30 min, washed twice using phosphate-buffered saline (PBS) solution (Sigma-Aldrich), then resuspended in 250 μl PBS and analyzed on a CytoFlex S flow cytometer (Beckman Coulter). Data were analyzed using FlowJo 10.8.1 software (BD Biosciences). Sample gating was standardized using CEM cells transfected with positive (SF2 strain Nef; NL4.3 strain Vpu) and negative controls (empty plasmids).

To quantify the relative function of each LTS-derived Nef or Vpu clone, the difference in median fluorescence intensity (MFI) of each antibody label was calculated between the GFP-positive (i.e., Nef/Vpu-transfected) and GFP-negative (*i.e.*, untransfected) cell gates. These values were then normalized to those of the positive control, which was examined in parallel, using the following formula: [(MFI_Neg_−MFIclone)]/[(MFI_Neg_−MFI_control_)]. A value of <1.0 indicates a relative function that is less than that of the positive control (SF2 for Nef or NL4.3 for Vpu), whereas a value of >1.0 indicates a relative function greater than that of the relevant control. Each Nef and Vpu clone was tested in at least three independent experiments, and results are reported as the mean.

### Mutagenesis

Participant-derived Nef or Vpu clones were subjected to site-directed mutagenesis to evaluate the impact of specific polymorphisms on protein function. Individual mutations were introduced into the parental gene sequence, which was then codon optimized (Codon Optimization tool; Integrated DNA Technologies) and synthesized commercially (eBlocks; Integrated DNA Technologies). Products were cloned into pSELECT-GFPzeo, sequence-validated and assessed for function as described. Codon optimized parental clones were used as controls. Each mutant clone was tested at least three times in independent experiments, and representative results are shown.

### Statistical Analysis

Statistical analyses were performed using Prism v9.0 (GraphPad). The Mann-Whitney *U*-test was used to compare Nef and Vpu functions in LTS and controls. It was also used to assess the relationship between population-level Nef and Vpu amino acid variation and protein function on a codon-by-codon basis, where multiple comparisons were addressed using a q-value approach (36). Fisher’s exact test was used to compare Nef and Vpu amino acid frequencies at specific residues between groups. Spearman’s correlation was used to analyze the relationship between *Nef* and *Vpu* functions within the same participant, as well as between protein functions and the participant’s clinical parameters (CD4+ T-cell count and pVL). An unpaired *t*-test was used to compare the function of the mutant to that of the parental clone.

### Data Availability

*Nef* and *vpu* sequences from LTS and a subset of *Nef* controls have been submitted to GenBank accession numbers ON044919-ON044988. Control *nef* sequences with accession numbers KC906733-KC907077 (28) and control *vpu* sequences with accession numbers MT116441-MT116772 (27) were deposited in GenBank previously. All clone sequences are provided in the Supplementary Data File; Tables S1 and S2. Full results of sequence-function analyses are provided in the Supplementary Data File; Tables S3-S6.

## RESULTS

### LTS and Control Study Participants

**FIGURE 1 ∣:**
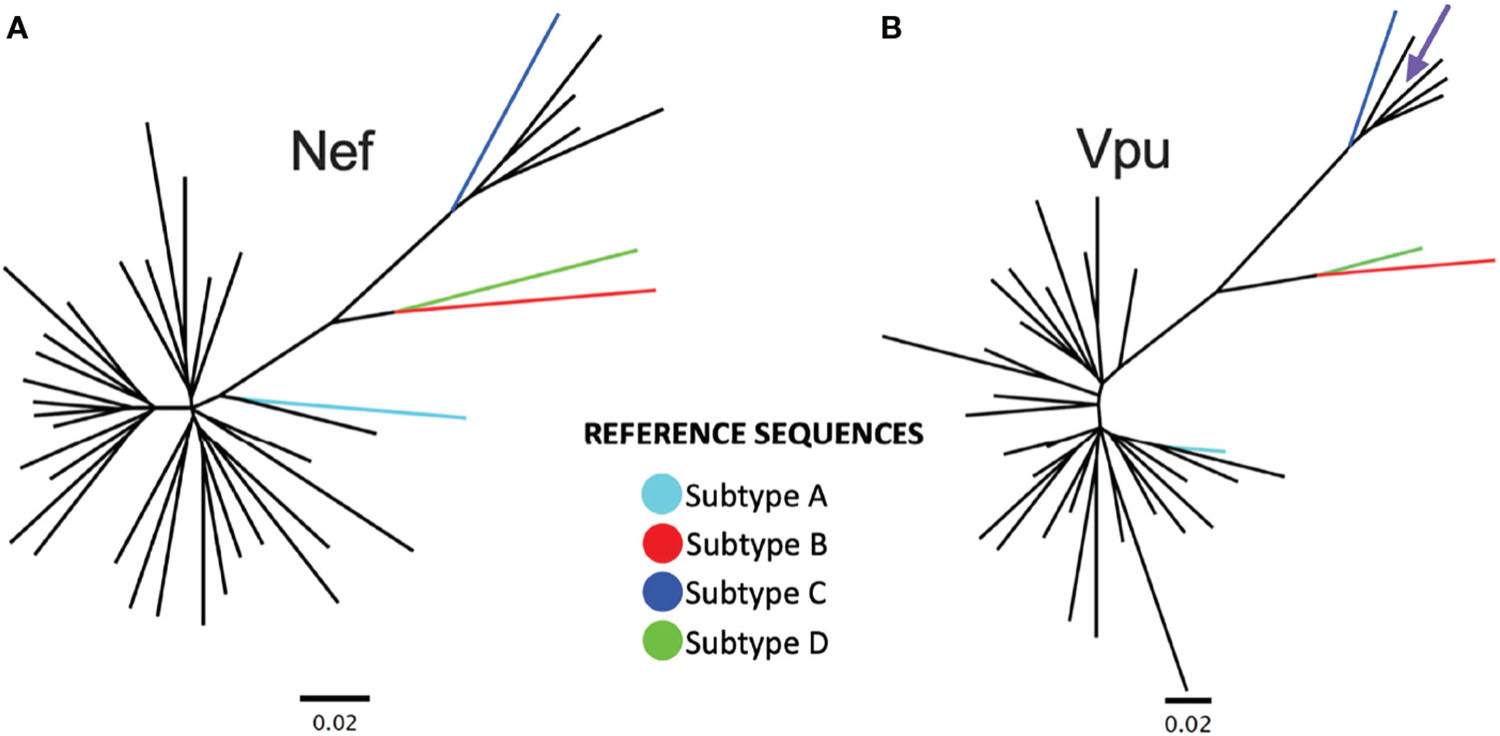
*Nef* and *Vpu* phylogenies from the survivor cohort. **(A)** Unrooted phylogeny inferred from an alignment comprising 34 *nef* sequences from Rwandan long-term survivors (LTS) along with HIV-1 subtype A, B, C and D reference sequences. The scale, shown below the phylogeny, is measured in estimated nucleotide substitutions per site. **(B)** A corresponding phylogeny for 34 *vpu* sequences. The arrow points to a *vpu* sequence with a branch length too short to see.

Our research group has promoted couples-based HIV counseling and testing in Rwanda since the mid-1980s (37). In 1994 however, the genocide against the Tutsi in Rwanda interrupted this work for nearly 6 years. Upon re-initiating the project, we identified 103 former clients who had been diagnosed with HIV-1 in 1986 and who had lived for more than a decade without access to cART. While their extended longevity fulfills a core criterion used to define the non-progressor phenotype, no other clinical information is available prior to the year 2000. To reflect this limitation, we refer to these individuals as long-term survivors (LTS). Since relatively few studies of HIV-1 non-progressors have been conducted in Africa, we were interested to explore the potential role of viral accessory protein function in this unique group. Plasma samples of varying quantity and quality, collected between 2000-2003, were available from 103 LTS, from which we were able to amplify paired *nef* and *vpu* sequences from 34 participants (Figure 1). As the majority of these clones (n=30 for *nef* and N=29 for *vpu*) were subtype A, we focused our subsequent functional analyses on the 29 LTS with subtype A *nef* and *vpu* sequences. These 29 LTS had remained healthy for a median [IQR] of 19.3 [15.9-24.6] years without cART (Table 1). As a comparator group, we used data from 166 clones (104 Nef and 62 Vpu) isolated from the plasma of 143 participants with chronic untreated HIV-1 subtype A infection (27, 28). Of these, 20 participants were enrolled in the Rwandan Heterosexual Transmission [HT] prospective study in Kigali, Rwanda from 2005-2009, and 123 participants were enrolled in the Uganda AIDS Rural Treatment Outcomes [UARTO] cohort in Mbarara, Uganda from 2002-2009 (Table 1).

### Phylogenetic Analysis of Nef and Vpu From LTS and Chronic Controls

**FIGURE 2 ∣:**
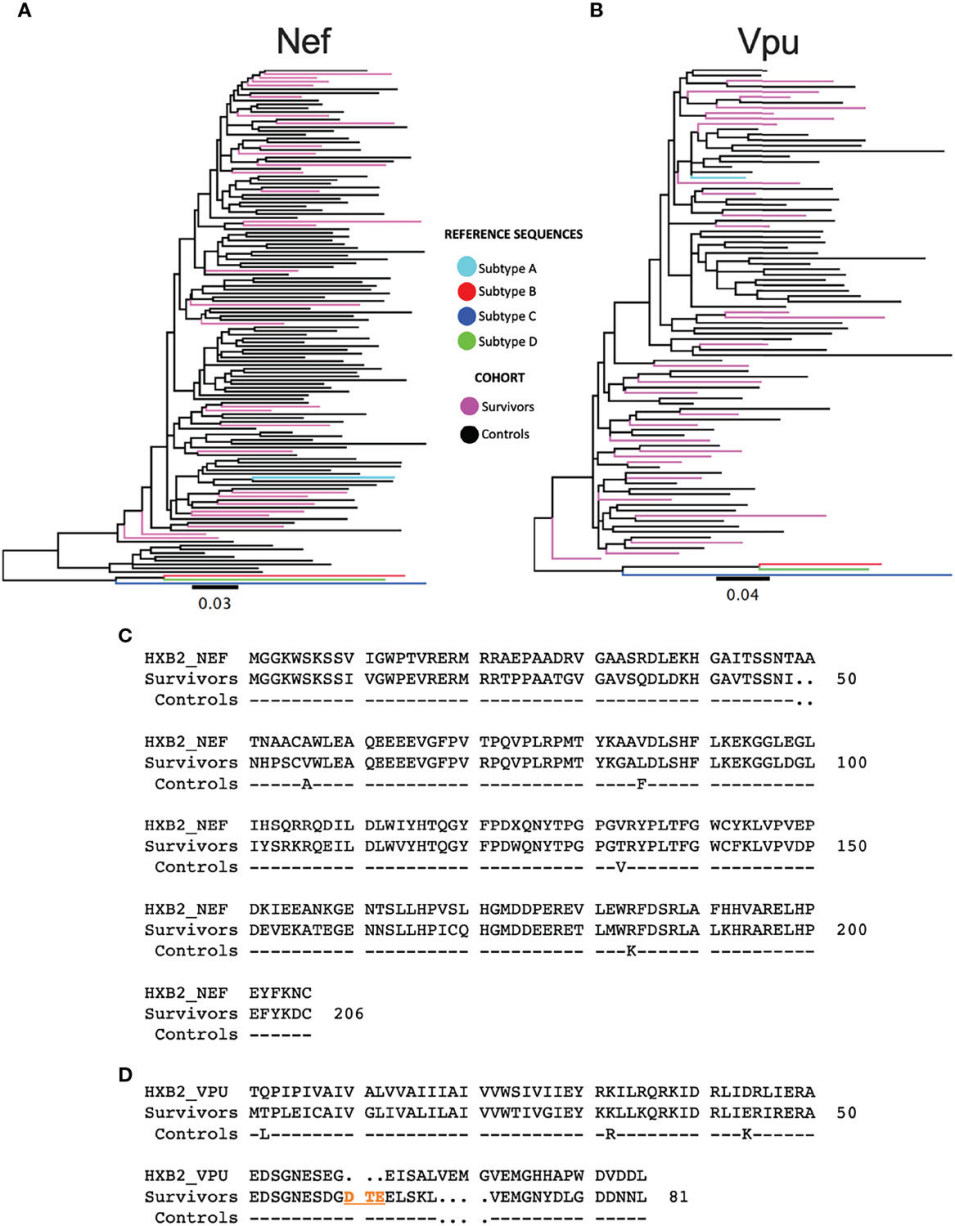
Phylogenetic trees of subtype A sequences from survivors and controls. **(A)** Phylogeny inferred from alignments of HIV-1 subtype A *nef* sequences from LTS and control participants, along with HIV-1 subtype **(A–D)** reference sequences. Sequences are colored by cohort (LTS in pink, controls in black). The tree is midpoint-rooted. Scale in estimated nucleotide substitutions per site. (B) Corresponding phylogeny for HIV-1 subtype A *vpu* sequences. **(C)** *nef* consensus sequences from LTS and control cohorts aligned to HXB2 subtype B reference. Residue numbering is based on HXB2. **(D)** *vpu* consensus sequences from LTS and control cohorts aligned to HXB2 subtype B reference. Residues 60-62 (DTE) shown in orange were present in the native subtype A sequences, but missing in HXB2. Likewise, HXB2 contains an insertion at residues 65-68, which were excluded from numbering. As such, the residue numbering for Vpu is based on the participant consensus.

Given the temporal and geographic variation between the LTS and control *nef* and *vpu* sequences, we first investigated these sequences for evidence of genetic clustering that could influence results interpretation. Maximum-likelihood phylogenies inferred from LTS and chronic *nef* and *vpu* sequence alignments however showed that LTS sequences interspersed relatively evenly among the chronic comparison sequences, with no overt segregation, supporting the latter as reasonable controls (Figures 2A, B). Some differences nevertheless existed between LTS and control consensus amino acid sequences, which differed at four of Nef’s 206 codons (56, 85, 133 and 184) and three of Vpu’s 81 codons (2, 32, and 44) (Figures 2C, D).

### Nef Function Is Reduced in LTS Clones

**FIGURE 3 ∣:**
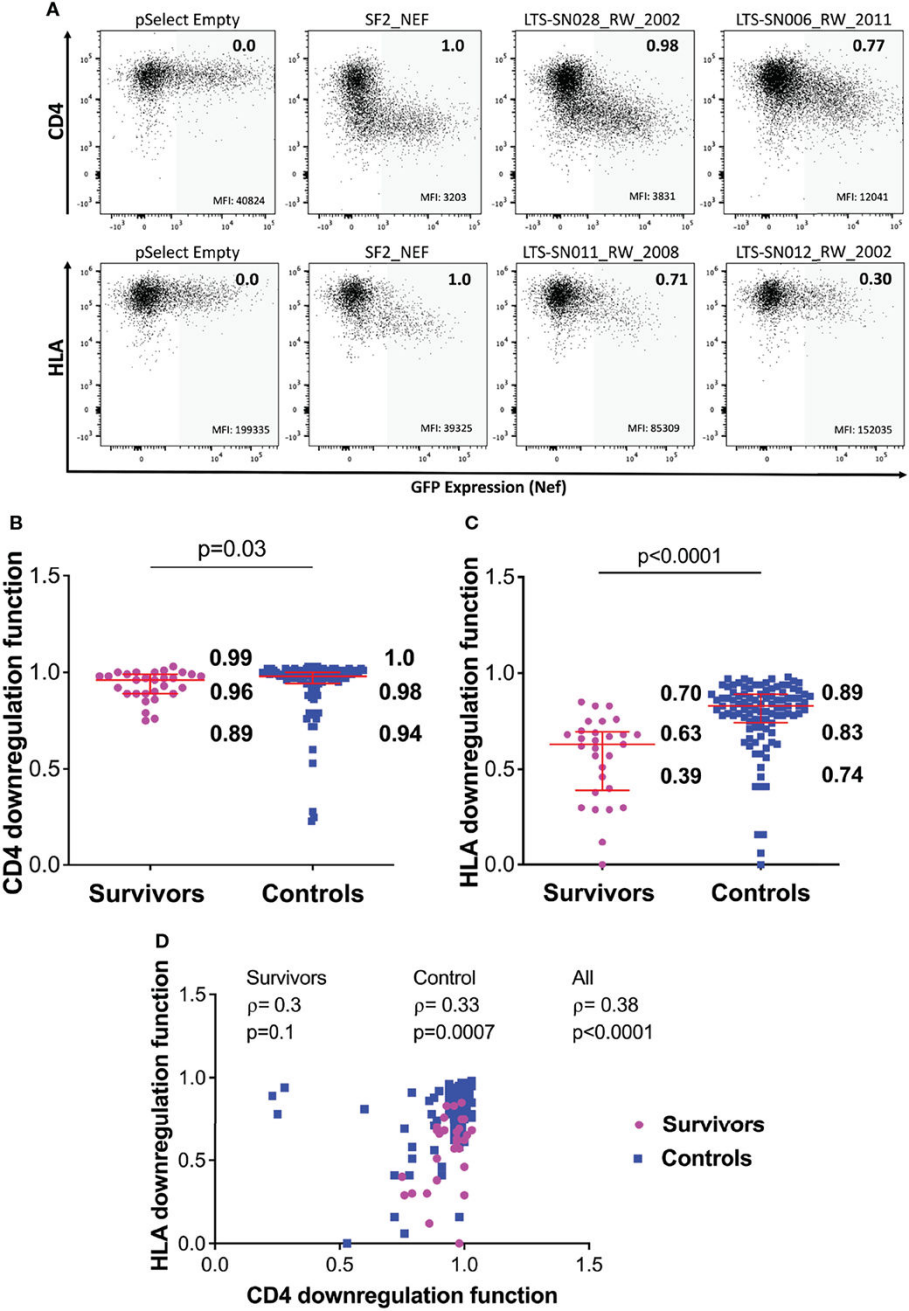
Nef-mediated CD4 and HLA downregulation function in survivors and controls. **(A)** Representative flow cytometry plots showing downregulation of CD4 (top) or HLA (bottom) following transfection of negative control (pSelect empty vector), positive control (SF2 Nef), a representative functional clone and a representative poorly-functional clone. Gray shading denotes the GFP-positive (Nef-expressing) gate that was used for quantification. Median fluorescence intensity (MFI) of receptor expression is indicated at the bottom of each gate, and the SF2-normalized downregulation function value (calculated as described in the Methods) is indicated in the top right of each plot. **(B)** Nef mediated CD4 downregulation functions of LTS and controls. Red box and whiskers denote the median and interquartile range, with values indicated on the plot. P-value is calculated using the Mann-Whitney U-test. **(C)** Comparison of Nef mediated HLA downregulation function among LTS and controls. **(D)** Relationship between Nef-mediated CD4 and HLA downregulation functions in individual and combined cohorts, assessed using Spearman’s correlation.

To assess whether Nef function is impaired in LTS, we examined Nef-mediated downregulation of CD4 and HLA class I using an established reporter cell assay where Nef clones are transfected into an immortalized CD4^+^ T-cell line that also expresses HLA-A*02 (representative raw data and normalized protein functions shown in Figure 3A). Each LTS Nef clone was examined in at least three independent experiments, with results reported as the mean of these measurements, while results for each Nef control clone is reported as the mean of two independent experiments (Supplementary Figure 2). Note that downregulation data for the 104 chronic Nef clones was obtained as part of our previously published study (28). To confirm the reproducibility of these historical data in the present context, we retested 25 chronic Nef clones from the previous dataset along with LTS clones and confirmed a high degree of correlation with no bias in the difference between historic and re-tested measures (Supplementary Figure 3).

Overall, Nef-mediated CD4 downregulation function was modestly lower among the 29 LTS clones compared to the 104 chronic clones: the normalized median activity of LTS clones was 0.96 (interquartile range [IQR] 0.89-0.99], while the median activity of chronic clones was 0.98 [IQR 0.94-1.0] (Mann-Whitney p=0.03; Figure 3B). Nef-mediated HLA class I downregulation was substantially lower among LTS clones: the normalized median activity of LTS clones was 0.63 [IQR 0.39-0.70], while the median activity of chronic clones was 0.83 [0.74-0.89] (Mann Whitney p<0.0001; Figure 3C). Moreover, and consistent with some prior studies, we found a weak positive correlation between these two Nef functions in the combined LTS and control data (ρ=0.38, p<0.0001), as well as within the control group (ρ=0.33, p=0.0007) (Figure 3D).

### Vpu Function Is Similar in LTS and Chronic Controls

**FIGURE 4 ∣:**
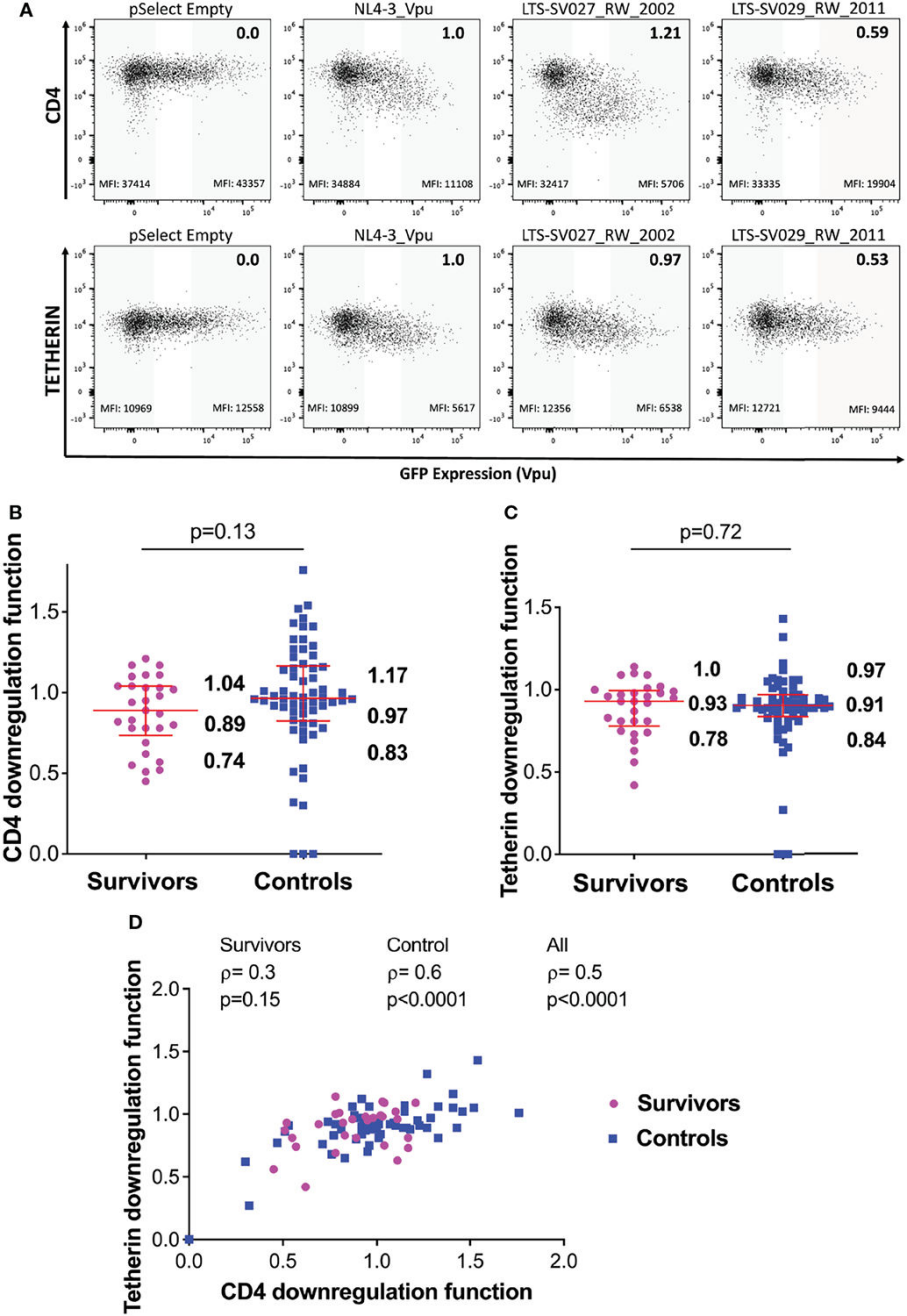
Vpu-mediated CD4 and Tetherin downregulation function in survivors and controls. **(A)** Representative flow cytometry plots showing downregulation of CD4 (top) or tetherin (bottom) following transfection of negative control (pSelect empty vector), positive control (NL4.3 Vpu), a representative functional clone and a representative poorly-functional clone. Gray shading at the left of each plot denotes the GFP-negative (untransfected) gate used for quantification, whereas grey shading at the right denotes the corresponding GFP-high (Vpu-expressing) gate. Median fluorescence intensity (MFI) of receptor expression is indicated at the bottom of each gate, and the NL4.3-normalized downregulation function (calculated as described in the Methods) is indicated in the top right of each plot. **(B)** Vpu mediated CD4 downregulation functions of LTS and controls. Red box and whiskers denote the median and interquartile range, with values indicated on the plot. P-value is calculated using the Mann-Whitney U-test. **(C)** Comparison of Vpu mediated Tetherin downregulation function among LTS and controls. **(D)** Relationship between Vpu-mediated CD4 and Tetherin downregulation functions in individual and combined cohorts, assessed using Spearman’s correlation.

To examine possible impairments in Vpu function in LTS, we used an established reporter cell assay where each Vpu clone was co-transfected with Rev into an immortalized CD4^+^T-cell line, and its ability to downregulate CD4 and Tetherin was measured by flow cytometry (representative raw data and normalized protein functions shown in Figure 4A). Both functions are reported as the mean of three independent replicate measurements (Supplementary Figure 4). Normalized Vpu function among the 29 LTS clones was compared to that of 62 Vpu clones previously isolated from individuals with chronic HIV-1 Subtype A infection (27).

Overall, the median Vpu-mediated CD4 downregulation function of the LTS clones was 0.89 [IQR 0.74-1.04], compared to 0.97 [IQR 0.83-1.17] in the chronic clones; however, this activity was variable within groups and the difference was not statistically significant (p=0.13) (Figure 4B). Similarly, median Tetherin downregulation function was 0.93 [IQR 0.78-1.0] in LTS clones, compared to 0.91 [IQR 0.84-0.97] in the control clones (p=0.72) (Figure 4C). We observed a positive correlation between these Vpu functions in our combined data from LTS and chronic clones (ρ=0.5, p<0.0001), as well as within the control group (ρ=0.6, p<0.0001) (Figure 4D).

### No Relationship Between Nef and Vpu Function in Paired LTS Clones

**FIGURE 5 ∣:**
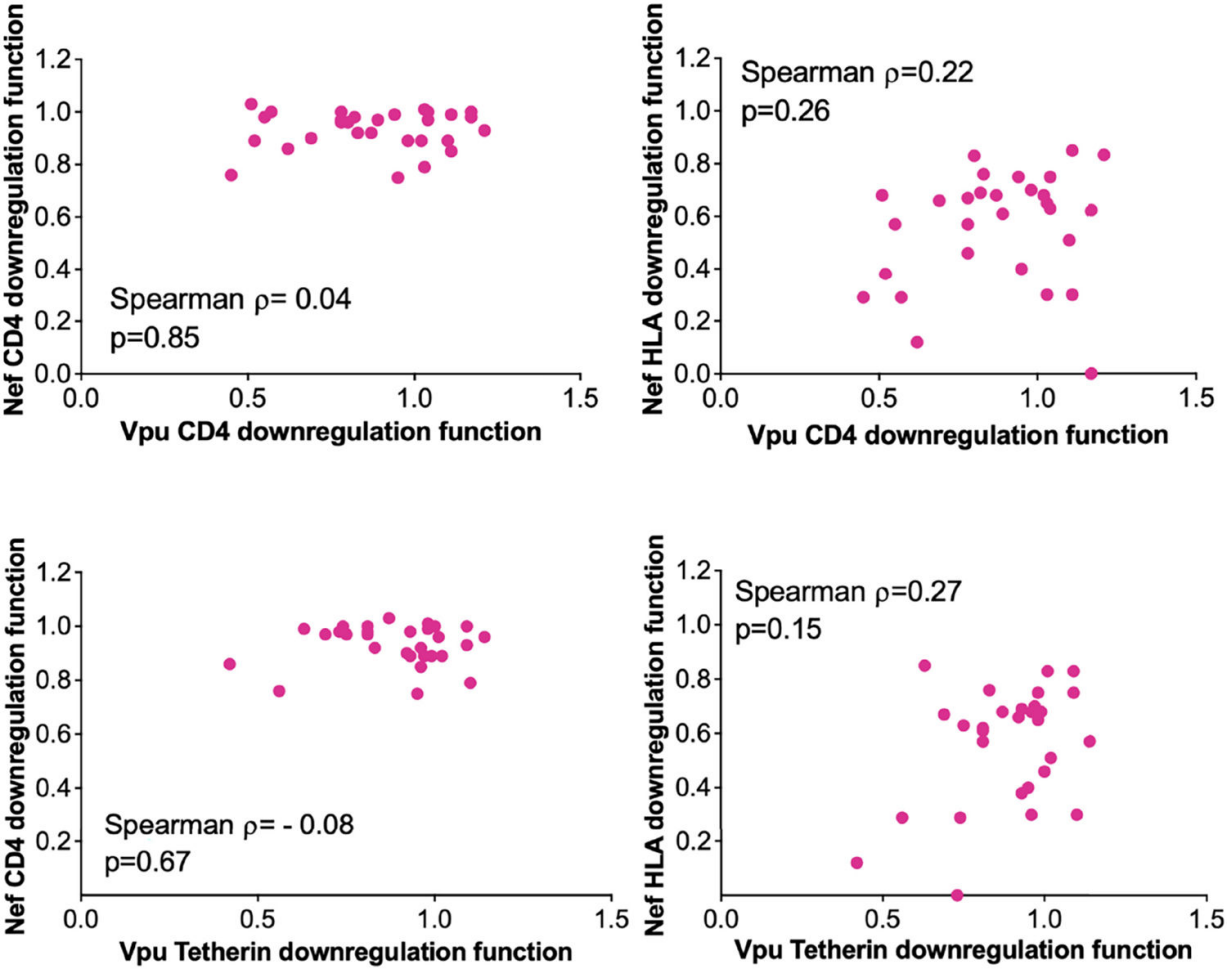
Relationship between within-host *Nef* and *Vpu* functions in the survivor cohort. Relationships are assessed using Spearman’s correlation.

Given their complementary immune evasion activities, within-host Nef and Vpu proteins could in theory compensate for impaired function in the other protein (12, 38, 39), in which case the function of within-host *nef* and *vpu* sequences would correlate inversely. Our analysis of paired Nef and Vpu clones from 29 LTS provided an opportunity to assess this. We observed no significant association between within-host Nef and Vpu functions however (Figure 5), suggesting that the activities of these viral proteins were largely independent.

### Associations Between Nef and Vpu Functions, and HIV Clinical Parameters

**FIGURE 6 ∣:**
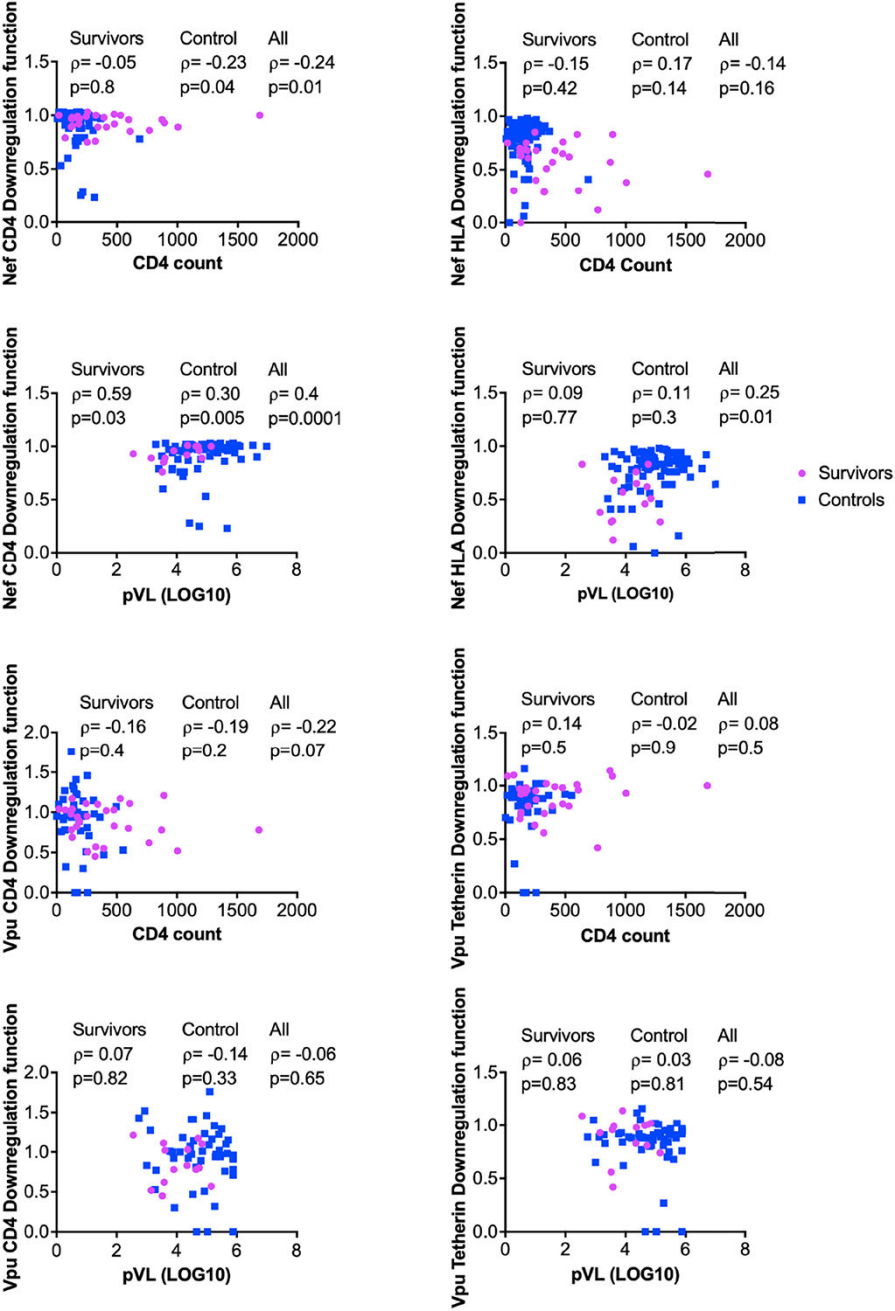
Relationship between *Nef* and *Vpu* functions and HIV clinical parameters (CD4 and pVL). Relationships were assessed within survivors, within controls, and in combined cohorts, using Spearman’s correlation.

The downregulation functions of Nef and Vpu enhance viral pathogenicity (14, 18, 22), and attenuated Nef and Vpu functions have previously been associated with slower disease progression (24, 26). We therefore analyzed the relationship between each Nef or Vpu function and clinical HIV-1 markers (CD4 cell count and plasma viral load (pVL)) (Figure 6). In combined data from LTS and control clones, Nef-mediated CD4 downregulation function correlated inversely with CD4 T cell count (ρ= −0.24, p=0.01) (Figure 6A) and positively with pVL (ρ=0.4, p=0.0001) (Figure 6C). The positive correlation between Nef-mediated CD4 downregulation function and pVL was also statistically significant when LTS and controls were analyzed separately (ρ= 0.59, p=0.03 and ρ= 0.3, p=0.005, respectively) (Figure 6C). In combined data from LTS and control clones, Nef-mediated HLA downregulation function also correlated positively with pVL (ρ=0.25, p=0.01) (Figure 6D); however, no association was observed between Nef-mediated HLA downregulation and CD4 cell count (Figure 6B). Similar associations between Nef function and clinical markers were typically not observed when LTS clones were analyzed alone, which may be due to limited statistical power, relatively lower function and/or less variable clinical phenotypes within this group. In contrast, no significant associations were found between Vpu function and HIV-1 clinical markers (Figures 6E-H). Overall, these observations support the notion that impaired Nef function is associated with slower disease progression.

### *Nef* and *Vpu* Sequence Polymorphisms Associated With Function

As previous studies have identified naturally occurring *nef* and *vpu* polymorphisms associated with downregulation function (27, 40, 41), we examined this question using linked sequence-function data from LTS and control participants. When aligning subtype A study sequences, we noted that Vpu residues 60-62 (typically DAE, DTE, or DTD), which were present in all of our sequences as well as most sequences from other subtypes published in the Los Alamos National Laboratory (LANL) HIV Sequence Database, were absent in the subtype B HXB2 reference sequence that is used to define the standard HIV-1 numbering convention. These residues were also absent in the NL4.3 Vpu clone that was used as a reference for functional assays. Furthermore, we noted that Vpu residues VEMG were duplicated in HXB2 and NL4.3 (at codons 65-68 and 69-72, respectively), whereas this did not occur in our native subtype A sequences (or those of other subtypes, based on LANL). To ensure that our sequence-function analysis of Vpu reflected natural sequence variation in subtype A, we kept residues 60-62 in our alignment and removed the duplicate VEMG motif at residues 65-68. The full Nef and Vpu alignments used in these analyses are provided in Supplementary Data File; Tables S1 and S2. The full results of our sequence-function analysis are shown in Supplementary Data File; Tables S3-S6; here, multiple comparisons were addressed using a false-discovery rate (q-value) approach (36).

Using a predefined significance threshold of p<0.05 and q<0.4, and a median functional difference of ≥ 5% between sequences having or lacking the amino acid of interest, we identified polymorphisms at 15 Nef codons that were associated with its HLA downregulation function (namely at residues 3, 9, 11, 14, 40, 56, 89, 105, 155, 157, 161, 184, 191, 196 and 206) (Supplementary Data File; Table S4). We also identified polymorphisms at seven of Vpu’s codons that were associated with its CD4 downregulation function: 2, 3, 5, 7, 45, 60 and 68 (Supplementary Data File; Table S5); as well as polymorphisms at nine of Vpu’s codons that were associated with its Tetherin downregulation function: 2, 26, 30, 58, 62, 68, 69, 70, 77 (Supplementary Data File; Table S6). We did not identify any residues associated with Nef-mediated CD4 downregulation function that met these criteria (Supplementary Data File; Table S3).

**FIGURE 7 ∣:**
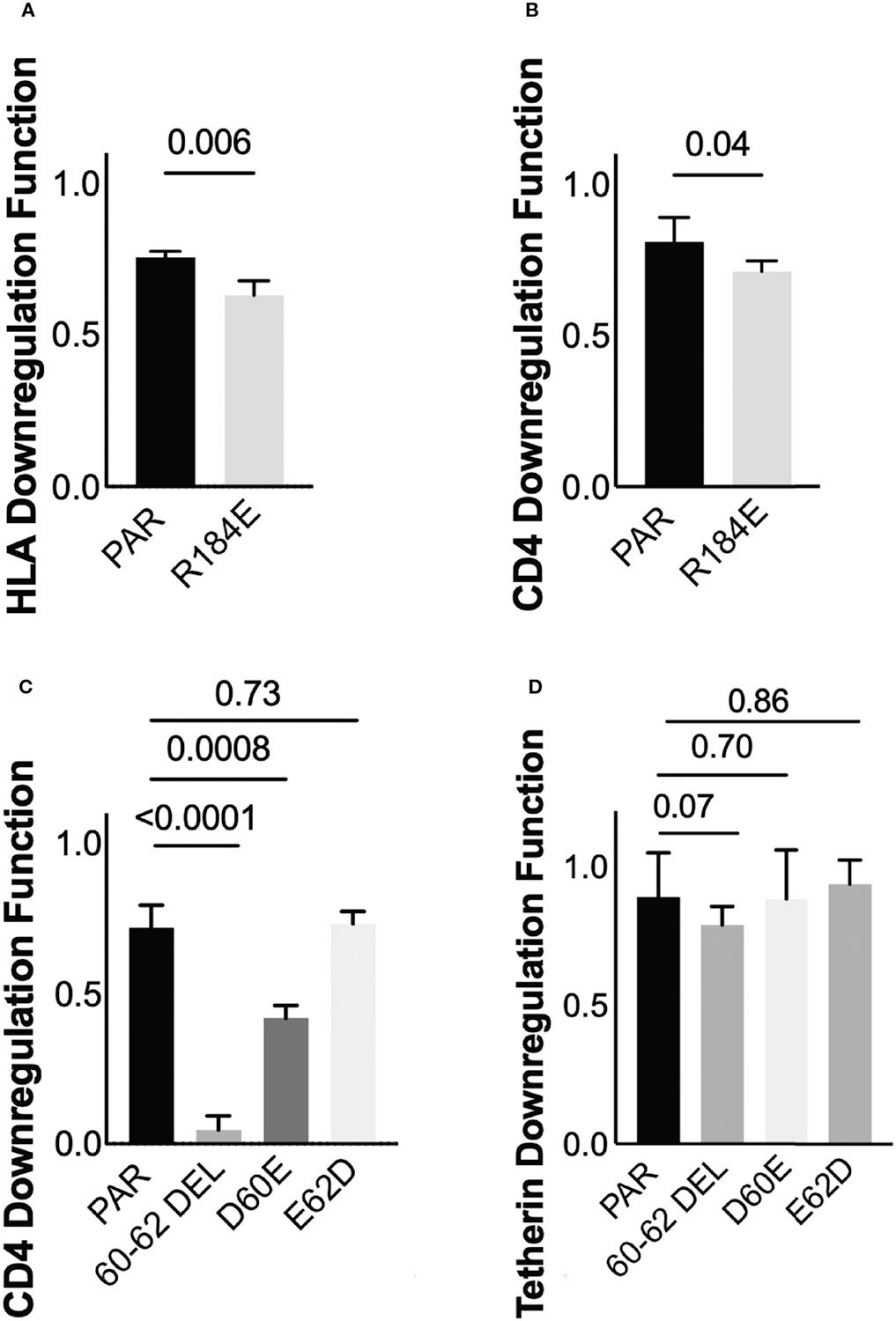
Verification of residues associated with *Nef* and *Vpu* functions **(A)** Nef-mediated HLA downregulation function of the parental (PAR) clone expressing R184, and the mutant harboring R184E. Results are expressed as mean of 3 replicate measurements with error bars denoting the median and interquartile range. **(B)** Same as A, but for Nef-mediated CD4 downregulation function **(C)** Vpu-mediated CD4 downregulation function of the parental (PAR) clone expressing D60 and E62, along with mutants harboring the 60-62 deletion, D60E or E62D substitutions **(D)** Same as C, but for Vpu-mediated Tetherin downregulation function.

Three of these polymorphisms occurred at codons where the population consensus differed between cohorts (Nef residues 56 and 184; Vpu residue 2; see Figures 2C, D). However, the distribution of residues Nef 56V/A and Vpu 2T/L did not differ significantly between cohorts (Fisher’s exact test, p=0.2 for Nef and p=0.16 for Vpu), so we did not pursue these further. Of the identified associations, we selected polymorphisms at Nef 184E, as well as Vpu 60D and Vpu 62E for *in vitro* validation. Nef 184E, a minority variant at this residue, was present in five (of 29; 17.2%) LTS clones compared to two (of 104; 1.9%) control clones (Fisher’s exact test, p=0.006), and was associated with impaired HLA downregulation function. Vpu 60D/E and 62D/E were of interest since they were located within the motif absent in HXB2 and NL4.3, and because the D60E substitution, an uncommon variant at this position, was associated with the single largest negative effect on Vpu-mediated CD4 downregulation function (−44%) in our study (Supplementary Data File; Table S5). To test the impact of Nef 184E, we synthesized codon-optimized clones based on isolate KC906874 from the control cohort, that encoded the parental 184R or the mutant R184E. We then compared the abilities of these clones to downregulate CD4 and HLA using our reporter cell assay in at least three replicate experiments. In agreement with our expectations, the R184E substitution significantly reduced Nef’s ability to downregulate HLA (p=0.006) (Figure 7A). We also observed that this mutation impaired CD4 downregulation compared to the parental clone (p=0.04) (Figure 7B).

Next, to test the impact of polymorphisms at Vpu codons 60 and 62, we synthesized codon-optimized clones based on isolate MT116708 from the control cohort that encoded the parental sequence, which featured D60 and E62. We also synthesized mutants encoding D60E or E62D, as well as a mutant that lacked residues 60-62 (consistent with NL4.3/HXB2). The relative abilities of each clone to downregulate CD4 and Tetherin were then assessed using our reporter cell assay in at least three replicate experiments. We observed that deletion of residues 60-62, located in the second alpha-helix of Vpu, abrogated CD4 downregulation function (p<0.0001) but reduced Tetherin downregulation function only modestly (p=0.07) (Figures 7C, D). In addition, consistent with our expectations, we found that D60E reduced CD4 downregulation function (p=0.0008), while E62D had no effect (p=0.73) (Figure 7C). Neither D60E nor E62D significantly altered Tetherin downregulation function compared to the parental clone (p=0.70 and p=0.86, respectively) (Figure 7D), though the modest enhancement by E62D was in line with our sequence/function analyses. Together, these observations indicate that Nef polymorphism 184E may attenuate function in an HIV-1 subtype A context. In addition, these findings suggest a potential role for Vpu residue 60 in modulating CD4 downregulation function in this subtype.

## CONCLUSION AND DISCUSSION

We investigated the sequence and function of HIV-1 accessory proteins Nef and Vpu in a unique cohort of 29 LTS individuals from Rwanda, who had survived with HIV-1 subtype A infection for a median of 19.3 years without antiretroviral treatment. LTS-derived clones did not display obvious genetic defects or deletions in Nef or Vpu that would be expected to affect their function. Furthermore, the sequences of LTS clones intermingled on a phylogenetic tree with those of 104 Nef and 62 Vpu clones from cART-naïve individuals living with HIV-1 subtype A from the same geographic region, suggesting that the LTS phenotype was not attributable to infection with a shared attenuated HIV-1 strain.

Overall, we observed that LTS Nef clones displayed significantly lower abilities to downregulate CD4 and HLA class I compared to control Nef clones. In contrast, both LTS and control Vpu clones downregulated CD4 and Tetherin to a similar degree. We also observed consistent positive correlations between Nef-mediated CD4 downregulation function *in vitro* and CD4 cell count as well as plasma viral load *in vivo*, while no similar correlation with HIV-1 clinical markers was found for Vpu. Together, these results suggest that impaired function of Nef, but not Vpu, contributes to slower progression in the LTS group; an observation that is consistent with prior studies from our group and others that found reduced Nef function in HIV-1 elite controllers and long-term non-progressors (24, 26, 42, 43).

In a linked sequence-function analysis of Nef, we identified polymorphisms at 15 Nef codons that were associated with differences in HLA downregulation, while no residues were associated with CD4 downregulation using pre-defined thresholds for significance. Of these, Nef 184E was found in a higher proportion of LTS clones (17.2%) versus control clones (1.9%), and introducing it into a participant Nef clone significantly reduced HLA and CD4 downregulation function, supporting the importance of this naturally arising polymorphism in modulating Nef function in HIV-1 subtype A. In a similar analysis of Vpu, we identified polymorphisms at seven and nine of Vpu’s codons that were associated with differences in CD4 and Tetherin downregulation, respectively. Of these, Vpu D60 and E62 were selected for further testing. Introducing D60E into a participant Vpu clone significantly reduced its ability to downregulate CD4 but not Tetherin, while introducing E62D did not affect downregulation of either protein. Notably, deletion of Vpu residues 60-62, which are present in most circulating HIV-1 sequences but absent in HXB2 and NL4.3, resulted in a near complete loss of CD4 downregulation activity, while Tetherin downregulation was largely unaffected. It is intriguing that NL4.3 Vpu remains highly functional despite lacking residues 60-62. We attribute this outcome to an unusual insertion that occurs at residues 65-68 only in the HXB2/NL4.3 lineage, which may compensate deletion of 60-62 by stabilizing alpha-helix 2, but additional studies will be needed to test this hypothesis. Even though Vpu function was not clearly associated with the LTS phenotype in our study, these findings nevertheless highlight polymorphic residues in Vpu that may contribute to modulation of CD4 downregulation function in HIV-1 subtype A, as well as other subtypes.

This retrospective study was restricted to individuals with a rare phenotype for whom viable specimens were available, which limited our statistical power to identify associations between sequence, function, and clinical markers of viral pathogenesis. The analyses of viral polymorphisms associated with protein function should be interpreted with caution, since observations based on individual clones may not be generalizable. In particular, the dataset did not allow us to perform an in-depth analysis of secondary mutations that might modulate the impact of Nef or Vpu polymorphisms; thus, additional studies of residues that covary with Nef 184E or Vpu 60D/62E could enhance efforts to identify functionally important motifs in these proteins. Furthermore, the lack of an association between Vpu and clinical markers should consider the relatively small size of the LTS group. Nevertheless, our final analysis of paired Nef and Vpu clones from 29 LTS is among the largest studies to examine HIV-1 accessory protein function in non-progressors. In addition, our assessment of LTS living with HIV-1 subtype A infection extends prior studies that looked only at subtype B. Given our focus on these two accessory proteins, we cannot rule out possible contributions of other viral genes to the LTS phenotype. Furthermore, we have not examined potential host genetic or immunological factors that may also result in relative control of HIV-1. Future studies will be needed to address these important issues, as well as investigate potential links between Nef function and reservoir size in long-term survivors living with non-B subtypes (44). Despite these limitations, our findings clearly indicate that impaired Nef function is associated with long-term survival in the context of HIV-1 subtype A.

## IMPLICATIONS

Understanding the host and viral factors that contribute to control of HIV-1 infection can improve our knowledge of the biology of viral pathogenesis and may also provide new insights to inform the design of vaccines or therapies to prevent, treat or possibly cure HIV. The results of this study indicate that impaired Nef function contributes to slower disease progression in African long-term survivors living with untreated HIV-1 subtype A infection. This observation is consistent with prior studies in the context of subtype B infection, and further supports ongoing efforts to target Nef using novel antiviral therapies. Moreover, our analyses identified natural polymorphisms that impair Nef and Vpu function, which may highlight critical motifs on these proteins that can serve as new targets for therapeutic development.

## Supplementary Material

Supplementary Data

Supplementary Figures

## Figures and Tables

**TABLE 1 ∣:** Characteristics of Rwandan long-term survivors and controls.

Characteristic	LTS cohort Vpu and Nef (subtype A) (N = 29)	Comparison cohort Vpu (subtype A) (N = 62)	Comparison cohort Nef (subtype A) (N = 104)
Female Sex, N (%)	29 (100)	34 (54.8)	56 (53.8)
Follow up in years before ART, median [IQR]	19.3 [15.9-24.6]	–	–
Age at cohort entry in years, median [IQR]	24 [23-27]	37 [32-40]	35 [30-40.5]
Country of origin (%)	Rwanda (100)	Rwanda (24.2); Uganda (75.8)	Rwanda (11.5); Uganda (88.5)
Most recent pVL pre-ART, median [IQR]	4.12 [3.55-4.72]	4.93 [4.41-5.45]	4.84 [4.42-5.53]
CD4 count at time of sampling, median [IQR]	320 [147-563]	154 [88-199]	170 [101-243]

## Data Availability

The datasets presented in this study can be found in Supplementary Material. Sequences are submitted to GenBank: accession numbers ON044919-ON044988 (for LTS clones); KC906733-KC907077 and MT116441-MT116772 (for control clones).
