# Supplementary material for: Attenuated HIV-1 Nef But Not Vpu Function in a Cohort of Rwandan Long-Term Survivors
Source: Front Virol. Author manuscript; Available in PMC 2022 Aug 17. (PMC9383652; doi:10.3389/fviro.2022.917902)
Supplement: Supplementary Figures [file NIHMS1828549-supplement-Supplementary_Figures.pdf]

Supplementary Figures for:

Attenuated HIV-1 Nef but not Vpu function in a cohort of Rwandan long-term survivors

Gisele Umviligihozo[1], Jaclyn K. Mann[2], Steven W. Jin[1], Francis M. Mwimanzi[1], Hua-Shiuan A. Hsieh[3], Hanwei Sudderuddin[4], Guinevere Q. Lee[5], Helen Byakwaga[6,7], Conrad Muzoora[5], Peter W. Hunt[6], Jeff N. Martin[6], Jessica E. Haberer[8,9], Etienne Karita[10], Susan Allen[11], Eric Hunter[11,12], Zabrina L. Brumme[1,4], Mark A. Brockman[1,3,4*]

[1] Faculty of Health Sciences, Simon Fraser University, Canada;

[2] HIV Pathogenesis Programme, University of KwaZulu-Natal, South Africa;

[3] Department of Molecular Biology and Biochemistry, Simon Fraser University, Canada;

[4] British Columbia Centre for Excellence in HIV/AIDS, Canada;

[7] Department of Medicine, Weill Cornell Medical College, USA;

[6] Mbarara University of Science and Technology, Uganda;

[7] University of California, San Francisco, USA;

[8] Center for Global Health, Massachusetts General Hospital, USA;

[9] Department of Medicine, Harvard Medical School, USA;

[10] Centre for Family Health Research, Rwanda;

[11] Department of Pathology and Laboratory Medicine, Emory University, USA

[12] Emory Vaccine Center at Yerkes National Primate Research Centre, USA

*** Correspondence:**
Professor Mark A. Brockman; mark_brockman@sfu.ca

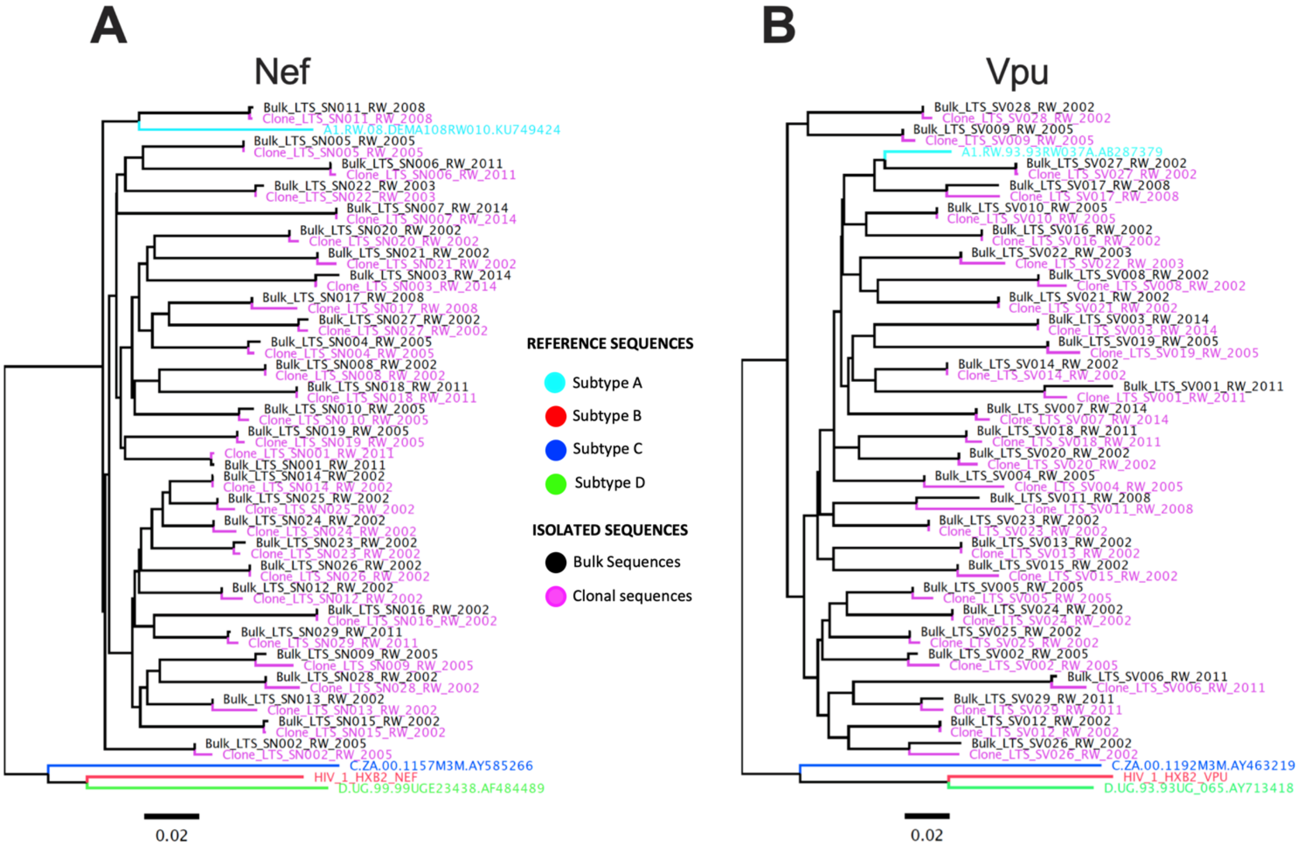

Supplementary Figure 1. Phylogenetic analyses verify that subtype A survivor clones cluster with their respective plasma sequences. *Panel A*: Maximum-likelihood phylogenetic tree inferred from HXB2-aligned Nef sequences derived from LTS plasma specimens (indicated as bulk; black text) and clonal sequences (magenta text). HIV-1 subtype A, B, C and D reference sequences (light blue, red, dark blue and green text, respectively) are included as comparators. *Panel B*: A similar phylogenetic tree inferred from HXB2-align Vpu sequences from LTS plasma (bulk; black text) and clones (magenta text). Both trees are midpoint rooted; scale displayed in estimated nucleotide substitutions per site.

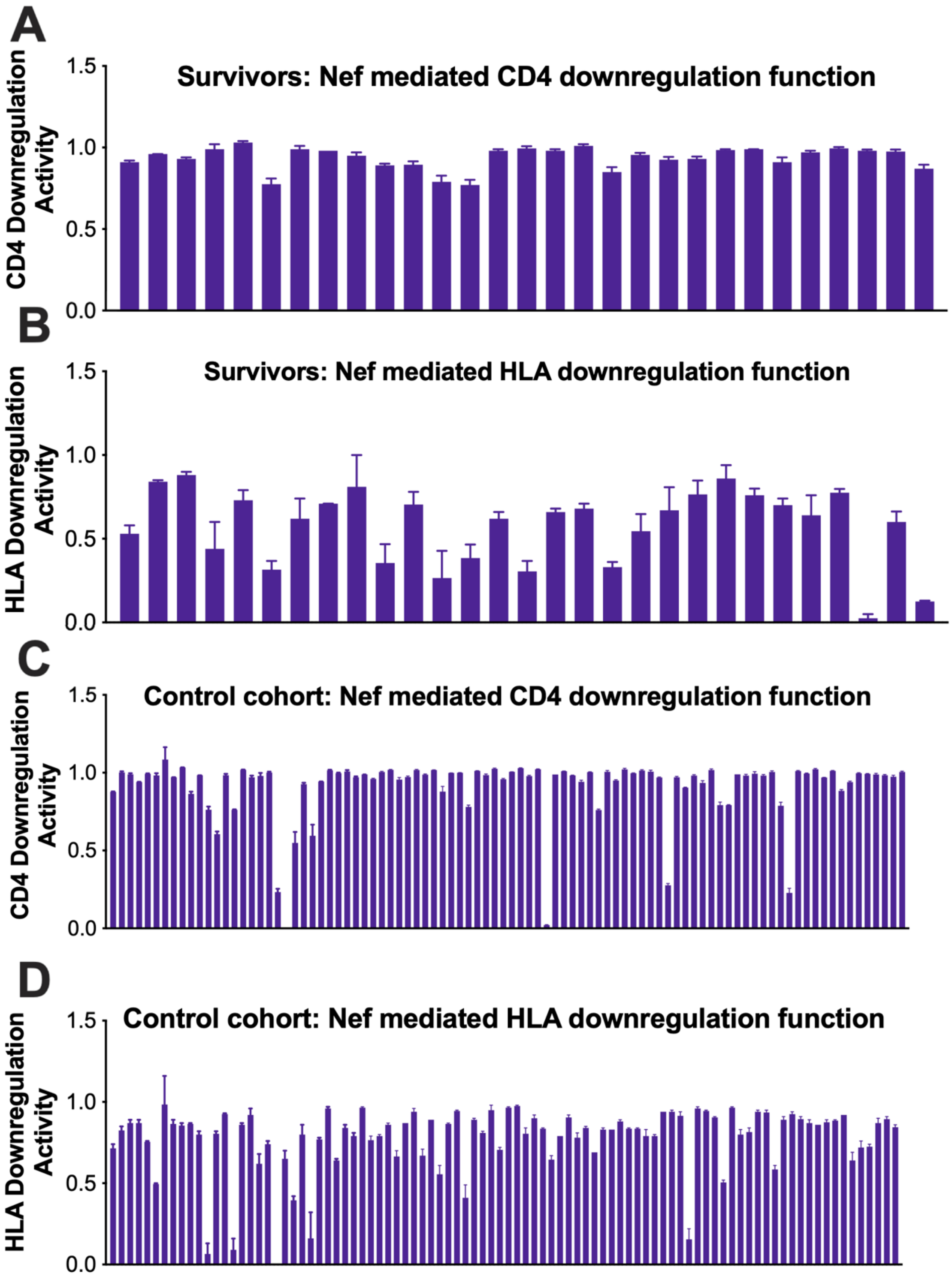

Supplementary Figure 2. Replicate values for Nef-mediated CD4 and HLA downregulation for survivor and control clones. Histogram and error bars denote median and standard deviation for a minimum of three independent replicate assessments per clone in the LTS group and the median and range of 2 replicate measurements in the controls.

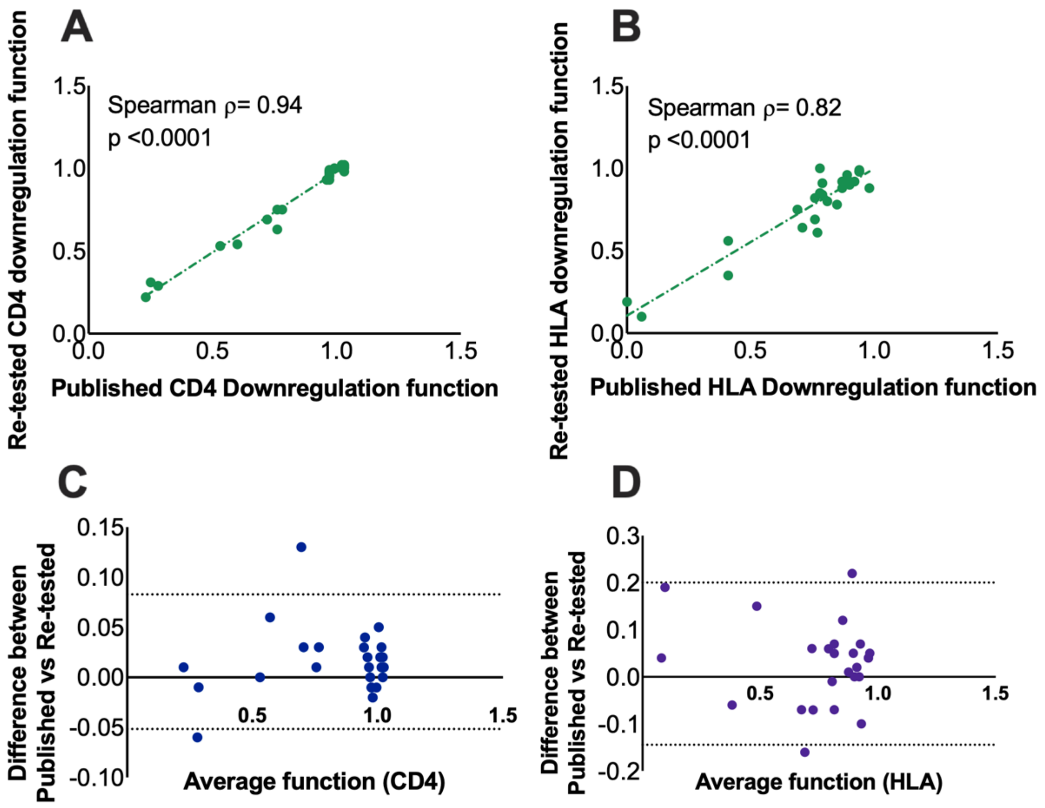

Supplementary Figure 3. Initial and re-tested values for *Nef* mediated CD4 and HLA downregulation functions for a random panel of 25 control samples. *Panel A*: Spearman's correlation between the published Nef-mediated CD4 downregulation values for 25 randomly selected control clones, and values re-tested for the present study. *Panel B*: Same as A, but for Nef-mediated HLA downregulation *Panel C*: Bland-Altman plot of the differences between the published and re-tested Nef-mediated CD4 downregulation values. Dotted lines denote ± 1.96 SD from the mean. *Panel D*: Same as C, for but for Nef-mediated HLA downregulation function.

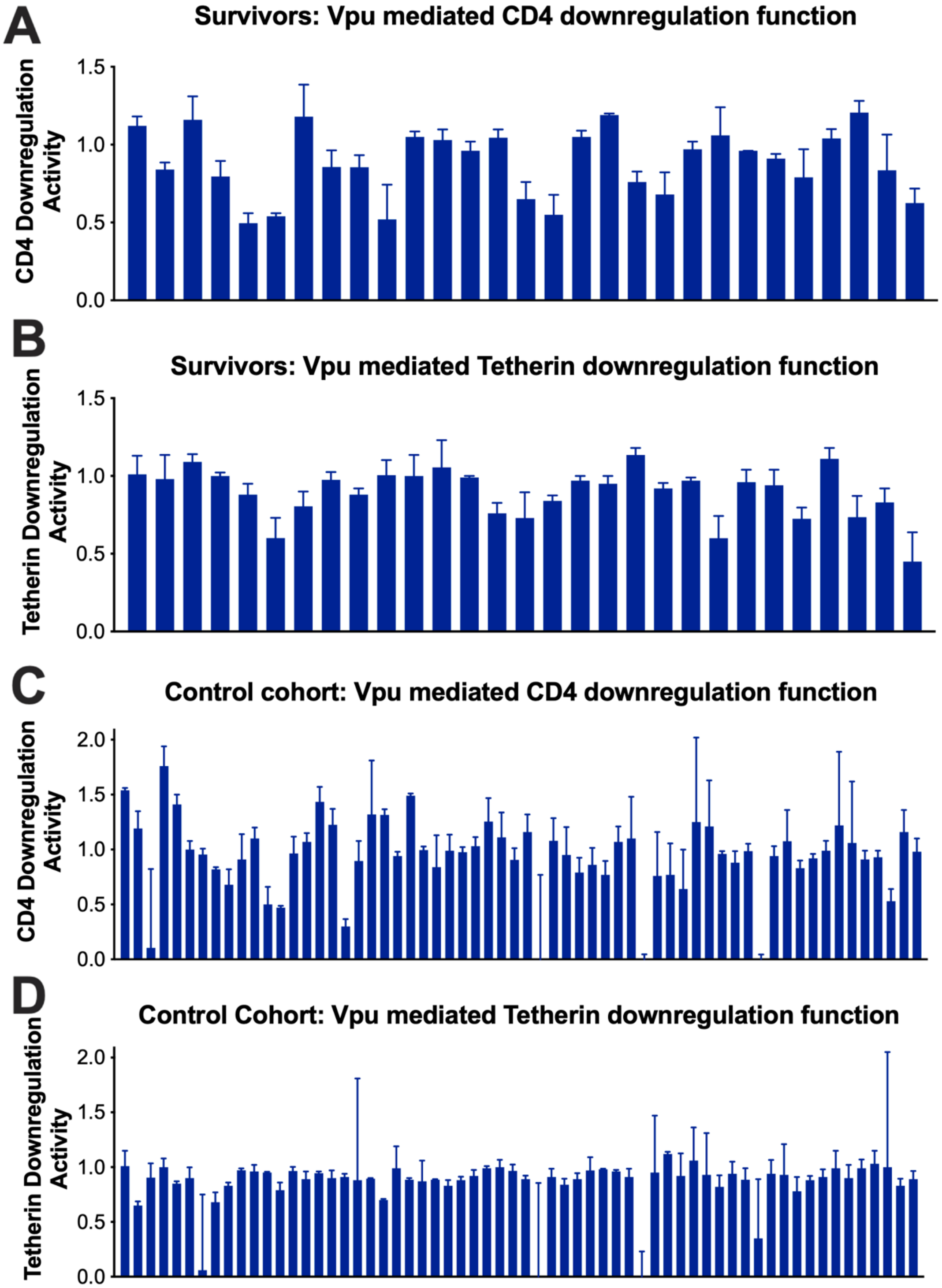

Supplementary Figure 4. Replicate values for Vpu-mediated CD4 and Tetherin downregulation for survivor and control clones. Histogram and error bars denote median and standard deviation for a minimum of three independent replicate assessments of each clone in the LTS group and the median and range of 2 replicate measurements in the controls.
